# Acquired drug resistance conferred by a KRAS gene mutation following the administration of cetuximab: a case report

**DOI:** 10.1186/1756-0500-6-508

**Published:** 2013-12-05

**Authors:** Hiroki Osumi, Satoshi Matsusaka, Eiji Shinozaki, Mitsukuni Suenaga, Mun Mingyon, Akio Saiura, Masashi Ueno, Nobuyuki Mizunuma, Toshiharu Yamaguchi

**Affiliations:** 1 Department of Gastroenterology, Cancer Institute Hospital of Japanese Foundation for Cancer Research, 3-8-31 Ariake, 135-8550, Koto-ku, Tokyo, Japan; 2 Surgery, Koto-ku, Japan; 3 Thoracic Center, Koto-ku, Japan

**Keywords:** KRAS, Acquired resistance, Cetuximab

## Abstract

**Background:**

Although a number of studies have reported acquired drug resistance due to administration of epidermal growth factor receptor antibody inhibitors, the underlying causes of this phenomenon remain unclear.

**Case presentation:**

Here we report a case of a 75-year-old man with liver metastasis at 3 years after a successful transverse colectomy to treat *KRAS* wild-type colorectal cancer. While initial administration of epidermal growth factor receptor inhibitors proved effective, continued use of the same treatment resulted in new peritoneal seeding. An acquired *KRAS* mutation was found in a resected tissue specimen from one such area. This mutation, possibly caused by administration of epidermal growth factor receptor inhibitors, appears to have conferred drug resistance.

**Conclusion:**

The present findings suggest that administration of epidermal growth factor receptor inhibitors results in an acquired *KRAS* mutation that confers drug resistance.

## Background

A better understanding of the mechanisms underlying changes in *KRAS* in response to epidermal growth factor receptor (EGFR) inhibitors might contribute to improvement in the treatment of colorectal cancer (CRC). Here we report a case of an acquired *KRAS* mutation that appears to have conferred drug resistance following administration of cetuximab and discuss it in light of the recent literature.

## Case presentation

The patient was a 75-year-old man with a history of hypertension, cerebrovascular disease, and adjustment disorder, but no known allergies. The patient’s father also had a history of colon cancer.

In December 2004, the patient underwent a transverse colectomy for transverse colon cancer (T3 N1 M0 stage IIIa) at another hospital. Due to adjustment disorder, the patient was closely monitored, but no adjuvant chemotherapy administered.

In March 2006, a colonoscopy revealed that the cancer had anastemotic recurrence, and in April 2006 the patient underwent a subtotal colectomy and anastomosis of the sigmoid colon and ileum.

In April 2007, the patient’s tumor markers were elevated and positron emission tomography-computed tomography (PET-CT) revealed metastasis to the liver and para-aortic lymph nodes. Chemotherapy with 5-fluorouracil/leucovorin/ oxaliplatin (FOLFOX4) was started as first-line treatment in May 2007. After 15 courses of treatment, the patient exhibited signs of progressive disease. Bevacizumab could not be administered, however, as the patient also had cerebrovascular disease.

In February 2008, chemotherapy with 5-fluorouracil/ leucovorin/irinotecan (FOLFIRI) was started as second-line treatment. After 16 courses of treatment, imaging revealed an increase in liver metastasis, and the patient was referred to our hospital.

**Figure 1:**
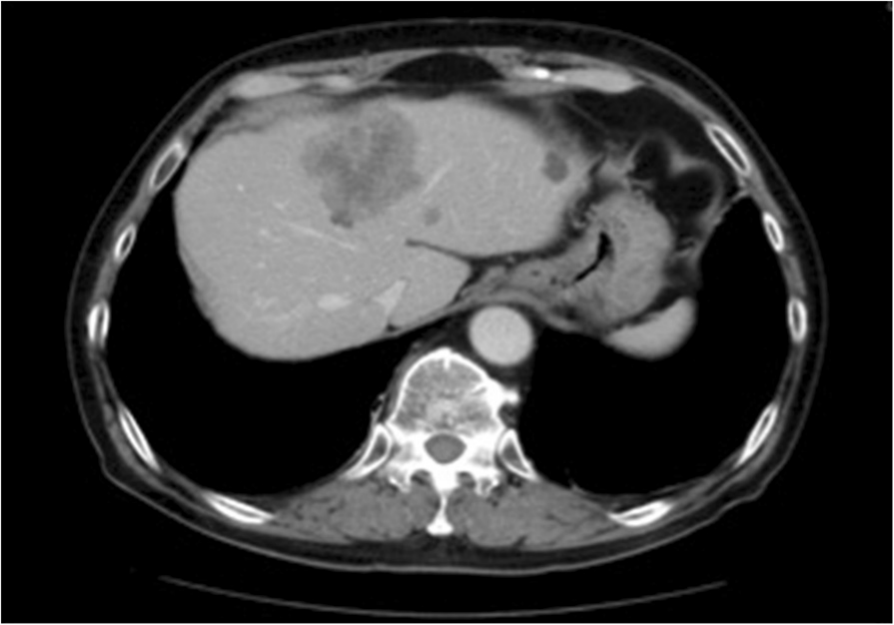
**Computed tomography of liver metastasis.** Abdominal CT showing shadows on liver at S4 and S8.

At the time of admission, the patient measured 186.5 cm in height, weighed 80.1 kg, had a Body Mass Index (BMI) of 23, and a Performance Status (PS) of 0. Normal heart sounds and breathing were noted, without abdominal abnormalities being observed, there was no leg edema, and the patient had grade1 peripheral neuropathy. Blood tests showed a normal blood count, and blood levels of carcinoembryonic antigen (CEA) and carbohydrate antigen (CA)19-9 at 88.8 ng/mL and 312.2 U/mL, respectively. Chest and abdominal X-rays, as well as an electrocardiogram, also revealed no abnormalities. An abdominal CT showed shadows on his liver at S4 and S8 (Figure 1). A PET-CT scan (1/29) showed liver metastases at S8, S4 and S3. The resected primary tumor tested positive for EGFR and showed the presence of wild-type *KRAS*. EGFR was analyzed by immunohistochemistry.

*KRAS* genotyping using tumor samples was analyzed by Luminex® assay.

The analytical sensitivity of the Luminex® assay is 0.4% (GENOSEARCH HS KRAS).

**Figure 2:**
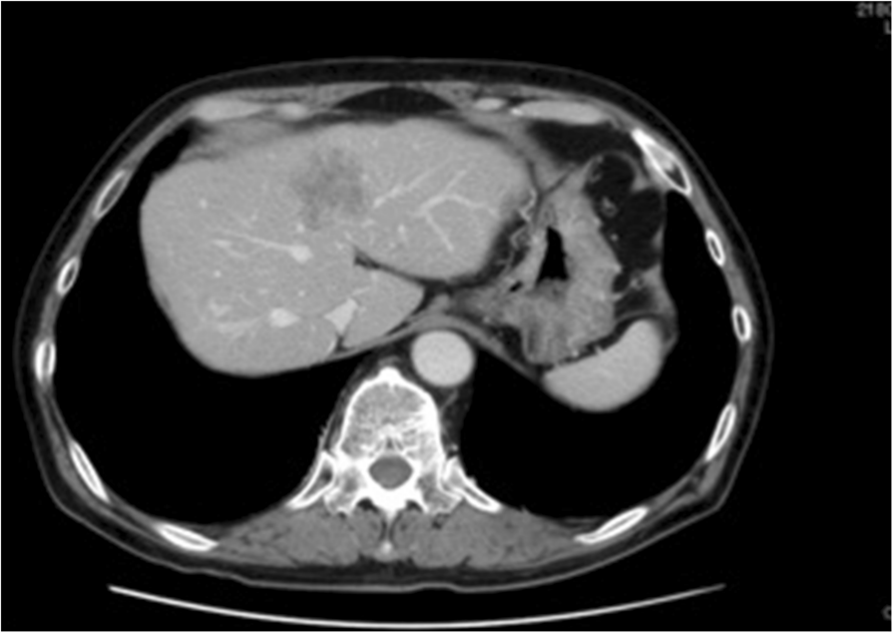
**Response of liver metastases to CPT-11-cetuximab.** After 13 courses of chemotherapy with irinotecan and cetuximab (CPT-11 + C-mab), blood levels of CEA and CA19-9 dropped to 14.3 ng/mL and 16.8 U/mL, respectively, and liver metastases showed reduction, indicating partial response.

After admission, chemotherapy with irinotecan and cetuximab (CPT-11 + C-mab) was started as third-line treatment in January 2009. After 13 courses of treatment, blood CEA and CA19-9 levels dropped to 14.3 ng/mL and 16.8 U/mL, respectively, and liver metastases showed a reduction (Figure 2), indicating a partial response. A left hepatectomy was performed in September 2009, and tumor markers dropped to normal levels. The resected tumor specimen showed the presence of wild-type *KRAS*.

In October 2009, postoperative chemotherapy was commenced with CPT-11 + C-mab. Six courses (courses 14–19) of treatment were completed, after which the patient only came in for follow-up without continuing chemotherapy.

In July 2010, the patient exhibited peritoneal dissemination and lung metastasis (S2,S4,S5,S8), and treatment with CPT-11 + C-mab was recommenced. After 18 courses of treatment (courses 20–37), no change was observed in lung metastases, and no new areas of metastasis were detected, indicating stable disease (SD).

In August 2011, the patient underwent lung metastasis resection. In October 2011, new areas of lung metastasis were found, while the primary tumor remained unchanged (SD). Treatment with CPT-11 + C-mab was started again (courses 38–48). Courses 39–41 and 45–48 consisted of only cetuximab. The lung metastases remained unchanged (SD) after 11 courses of treatment.

**Figure 3:**
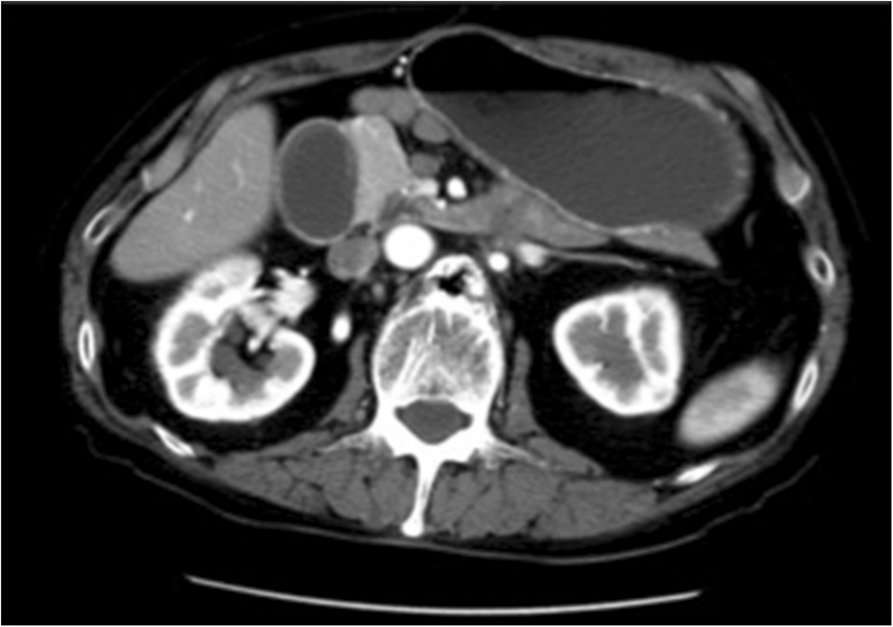
**Obstruction in ileum due to seeding in duodenum.** In May 2012, patient had obstruction in ileum due to seeding in duodenum. Emergency gastric bypass surgery was performed. KRAS mutation was detected in resected seeding tissue.

In May 2012, emergency gastric bypass surgery was performed for an obstruction in the ileum due to seeding in the patient’s duodenum (Figure 3). A *KRAS* mutation was detected in the resected seeding tissue.

At present, only palliative treatment is being administered as the patient has completed standard treatment.

While the patient’s resected tumors from both 2004 and 2009 showed the presence of wild-type *KRAS*, and the administration of cetuximab for 2 years and 4 months proved effective, the resected seeding tissue from his intestinal obstruction was found to have a *KRAS* mutation. Hence, it can be possibly concluded that administration of EGFR inhibitors resulted in an acquired *KRAS* mutation that conferred drug resistance.

## Conclusions

In 1988, Vogelstein et al*.* proposed a multi-stage theory of carcinogenesis known as the adenoma-carcinoma sequence, in which colorectal cancer arises due to mutations that activate multiple oncogenes and inactivate tumor suppressor genes, which then accumulate on the epithelium of a normal colon, forming adenomas. *KRAS* mutations were proposed to be driver mutations in colorectal carcinogenesis [1]. Earlier studies have noted similar *KRAS* mutations in both the primary tumor and metastases in more than 90% of patients with CRC or lung cancer [2,3]. Moreover, in addition to these two studies, another report also noted a small number of cases of *KRAS* mutations in metastases arising from wild-type *KRAS* primary tumors [4]. Colorectal tumors with wild-type *KRAS* are often sensitive to EGFR blockade [5]. The mechanism underlying an acquired resistance to EGFR inhibitors, however, remains largely unknown. The previously reported lower concordance levels of *KRAS* between the primary tumor and metastases are likely due to bias arising from false-negative results in underpowered studies and the correct evaluation of the amount of tumor tissue in the sample, or the sensitivity of the testing method used [6]. Previously published data showed that a considerable fraction of colorectal lymph node metastases do not resemble the primary tumor in terms of *KRAS* mutation status [4]. Heterogeneity in lymph node metastases could explain this discordance in a small number of cases, but its main mechanism is unknown. Misale et al. also reported that use of anti-EGFR drugs for metastatic CRC contributed to acquisition of a *KRAS* mutation [7]. There are two possible explanations for the discordant results for *KRAS*: heterogeneity of *KRAS* status within the primary tumor [8-10], or clonal selection during the process of metastasis [6]. Another clinical study identified wild-type *KRAS* in 10 patients with acquired resistance to anti-EGFR therapy. Meanwhile, a *KRAS* G13D mutation was identified in 4 further cases, and the simultaneous presence of G12D and G13D mutations in one. In 6 patients for whom sufficient pre-treatment tumor samples were available for high-coverage 454 sequence analyses or beads, emulsion, amplification and magnetics, *KRAS* mutations were found to be absent. Tumors from a further 8 patients treated with cytotoxic chemotherapy but not previously exposed to anti-EGFR therapies were also analyzed by 454 deep sequencing. In all 8 cases, the analyses identified no evidence of a *KRAS* mutation. These results indicate an association between treatment with anti-EGFR antibodies, not cytotoxic chemotherapy, and acquisition of *KRAS* mutations [7]. The mechanism of resistance to anti-EGFR therapy has recently been clarified. Montagut et al. reported an acquired EGFR ectodomain mutation (S492R) that prevented cetuximab binding, thus conferring resistance to this drug. The cells with this mutation, however, retained binding to panitumumab, which inhibited their growth. In that study, 2 of 10 patients acquired this mutation after cetuximab treatment. One patient with cetuximab resistance and harboring the S492R mutation responded to treatment with panitumumab. This indicates that panitumumab may be effective in patients with the S492R mutation after failure of cetuximab. A re-biopsy would be needed before commencing treatment with a molecularly targeted drug, however [10,11]. In further study, we aim to analyze *KRAS* status by using circulating tumor cells, which play a core role in liquid biopsy. Resistant cells remained sensitive to combinatorial inhibition of EGFR and mitogen-activated protein-kinase (MEK), and mutated *KRAS* alleles were detected in the blood of cetuximab-treated patients as early as 10 months before radiographic documentation of disease progression [7].

In summary, there is a growing body of evidence to suggest that EGFR inhibitor-induced *KRAS* mutations that associates with tumor recurrence. The results of the present study suggest that such mutations could be identified non-invasively months before disease progression became evident radiographically, and that early initiation of a MEK inhibitor would be a rational strategy for delaying or reversing drug resistance. If such strategies were employed, this might allow ineffective drugs to be terminated earlier and more effective treatment strategies pursued.

## Consent

Written informed consent was obtained from the patient for publication of this case report and accompanying images. A copy of the written consent is available for review by the Editor-in-Chief of this journal.

## Abbreviations

CT: Computed tomography; PET-CT: Positron emission tomography-computed-tomograpy; Bev: BGevacizumab; BMI: Body mass index; PS: Performance status; CEA: Carcinoembryonic antigen; CA19-9: Carbohydrate antigen; BEAMing: Beads, emulsion, amplification and magnetics; CTCs: Circulating tumor cells.

## Competing interests

The authors declare that they have no competing interests.

## Authors’ contributions

The original manuscript was written by HO. HO and MS and ES and MS and NM performed chemotherapy for mCRC. AS and MU and TY performed a left hepatectomy, MM performed a lung resection. All authors contributed to drafting and editing the manuscript. All authors read and approved the final manuscript.
